# pSIR-bsr, a self-inactivating retrovirus vector expressing the blasticidin S-resistance gene

**DOI:** 10.1093/biomethods/bpab022

**Published:** 2021-12-06

**Authors:** Hodaka Fujii

**Affiliations:** Department of Biochemistry and Genome Biology, Hirosaki University Graduate School of Medicine, Hirosaki, Aomori 036-8562, Japan

**Keywords:** blasticidin S, blasticidin S-resistance gene, bsr, self-inactivating retrovirus

## Abstract

Self-inactivating retrovirus vectors are useful tools for generating stable cell lines harbouring designed exogenous sequences but lacking the constitutive transcriptional activity of the long terminal repeats that are usually retained by non-self-inactivating retrovirus vectors. Thus, self-inactivating retrovirus vectors are ideal vehicles for integrated transgenes comprising transcriptional regulatory sequences, and for the genes expressed by these regulatory sequences. This article describes the development of a self-inactivating retrovirus vector retaining a blasticidin S-resistance (*bsr*) gene. The vector, named pSIR-bsr, would be useful for transducing multiple expression vectors with different selection markers.

## Introduction

Retroviral vectors are used widely to generate transformed cells via stable integration of exogenous DNA sequences. Retroviral vectors retain transcriptionally active long terminal repeats (LTRs) for expression of their genomic RNA. The retroviral genomic RNAs are converted into genomic DNA by viral reverse transcriptase. Since the wild-type LTR contains enhancer sequences, it can drive constitutive transcription of a gene inserted downstream of the 5ʹ-LTR. By introducing a deletion mutation into the 3ʹ-LTR, which removes the enhancer sequences, the 3ʹ-LTR is copied and replaces the 5ʹ-LTR after reverse transcription, thereby shutting down the promoter activity of the 5ʹ-LTR [1–5]. These self-inactivating retrovirus vectors are useful tools for generating stable cell lines harboring designed exogenous sequences in the absence of the constitutive transcriptional activity of the LTR, which is usually retained by non-self-inactivating retrovirus vectors. This makes self-inactivating retrovirus vectors ideal hosts for integrated transgenes comprising transcriptional regulatory sequences, and for the genes that they express.

My research group has used self-inactivating retrovirus vectors for transduction of gBlock, which is a U6 promoter-driven guide RNA. This was used for engineered DNA-binding molecule-mediated chromatin immunoprecipitation using the clustered regularly interspaced short palindromic repeats system [6]. However, not many self-inactivating retrovirus vectors harbor a drug resistance gene for selection. For example, to the best of my knowledge, only one vector with a neomycin-resistance gene (pSIR-neo) is available through Addgene. Some widely used cell lines such as the 293T retain the neomycin-resistance gene in their genome; therefore, this vector cannot be used for these cells. This means that self-inactivating retrovirus vectors harboring different drug resistance genes are needed.

Here, I developed a self-inactivating retrovirus vector retaining the blasticidin S-resistance (*bsr*) gene. This vector, called pSIR-bsr, would be useful for transduction of multiple expression vectors with different selection markers.

## Materials and methods

### Plasmids

To construct the pSIR-bsr plasmid, the coding sequence of the blasticidin S deaminase gene (the *bsr* gene) [7] was synthesized and cloned into the pEX-A2J2 vector (Eurofins Genomics, Tokyo, Japan). The synthesized DNA was used as a template for polymerase chain reaction (PCR) amplification using pSIR-bsr sense (28764: 5ʹ-aagatcgatctgatcaccATGAAAACATTTAACATTTCTCAAC-3ʹ) and pSIR-bsr antisense (28765: 5ʹ-gaggatcatccagccTTAATTTCGGGTATATTTGAGTGGA-3ʹ) primers. The PCR product was cloned into pSIR (Clontech Laboratories, Mountain View, CA, USA) digested with Bcl I and Nae I using the Gibson Assembly procedure and a Gibson Assembly kit (E2611S; New England Biolabs, Ipswich, MA, USA). The sequences of the cloned fragment were confirmed by Sanger sequencing using the above-mentioned primers. The Addgene plasmid no. of pSIR-bsr is 176507.

### Cell lines

The 293T cell line was derived by transformation of human embryonic kidney 293 cells using the SV40 large T antigen [8]. The 293T and 293T-derived cells were cultured in Dulbecco’s Modified Eagle Medium (DMEM) (FUJIFILM Wako Pure Chemical, Osaka, Japan) supplemented with 10% fetal calf serum (FCS).

### Transduction of the retroviral plasmid

For transduction of pSIR-bsr into 293T cells, 5.5 µg of the plasmid was transfected into 1 × 10^6^ of 293T cells along with 5.5 µg of pPAM3, a helper plasmid that expresses virus components necessary for the production of retrovirus particles [9], using Lipofectamine 3000 (Thermo Fisher Scientific, Waltham, MA, USA). Two days after transfection, the viral supernatant was harvested and used to infect 293T cells. Infected cells were selected in culture medium containing blasticidin S (4.5 µg/ml; FUJIFILM Wako Pure Chemical).

### Genomic PCR

Genomic DNA was purified from 293T and 293T-derived cells using a DNeasy Blood & Tissue Kit (69504; QIAGEN GmbH, Hilden, Germany) and subjected to PCR with EmeraldAmp PCR master mix (RR300, Takara Bio, Shiga, Japan). Primers pSIR-bsr sense (28764) and pSIR-bsr antisense (28765) were used to amplify the *bsr* gene. The PCR conditions were as follows: 94°C for 2 min; followed by 35 cycles of 94°C for 30 s, 60°C for 20 s and 72°C for 45 s; with a final extension at 70°C for 10 min. Amplicons were analysed by electrophoresis in 1% agarose gels.

### Cell proliferation assay

The 293T cells and 293 T cells (1 × 10^5^) transduced with pSIR-bsr were plated into the wells of a six-well tissue culture dish (in triplicate) and cultured in DMEM/10% FCS containing 4.5 µg/ml blasticidin S. Cells were detached from the wells by incubation with 1 ml trypsin (0.5 g/l)/ethylenediaminetetraacetic acid (EDTA) (0.53 mmol/l) (35553-74; Nacalai Tesque, Kyoto, Japan) at 37°C for 5 min. The trypsin was neutralized with culture medium and then centrifuged at 300*g* for 5 min at room temperature. After removing the supernatant, cells were suspended in culture medium and counted using the TC20^TM^ Automated Cell Counter (Bio-Rad Laboratories, Inc., Hercules, CA, USA). After plating the cells in a six-well tissue culture dish (in triplicate), cell numbers were counted on Days 2, 5 and 8. Cells (1 × 10^5^) were replated after counting on Days 2 and 5.

### Statistical analysis

Statistical differences in cell numbers in the proliferation assay were analysed using the *t*-test.

## Results and discussion

**Figure 1:**
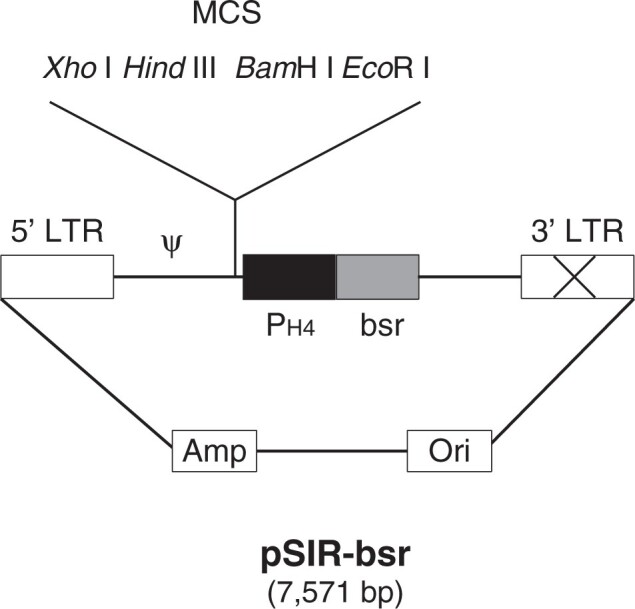
A schematic representation of the pSIR-bsr plasmid. The 3ʹ-LTR contains a 176-bp deletion, which removes enhancer sequences. Amp, ampicillin resistance gene; Ori, replication origin; 𝜓, packaging signal; MCS, multicloning site.

To increase the flexibility of selection markers for self-inactivating retroviral vectors, the pSIR-bsr plasmid expressing the *bsr* gene under control of the histone H4 promoter was constructed (Fig. 1).

**Figure 2:**
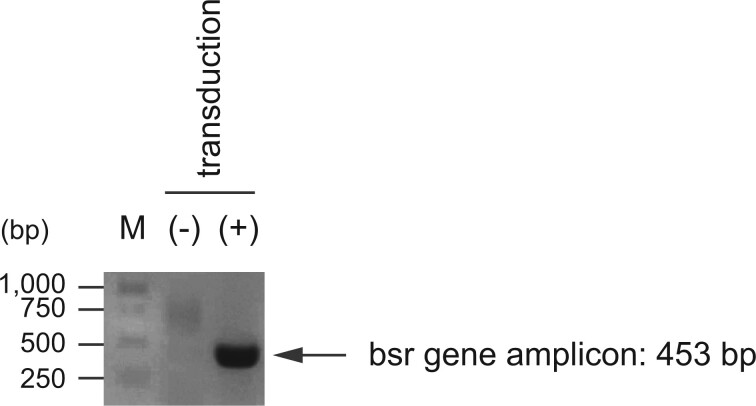
PCR amplification of the *bsr* gene from genomic DNA extracted from the parental 293 T cells (–) and 293 T cells transduced with pSIR-bsr (+). The expected band size is 453 bp.

The retroviral plasmid was then transduced into 293T cells, which were then selected with blasticidin S. Genomic DNA was purified from selected cells and subjected to PCR using primers specific for the *bsr* gene. Amplicons of the *bsr* gene were detected in genomic DNA from blasticidin S-selected cells but not in genomic DNA from parental 293T cells (Fig. 2).

**Figure 3:**
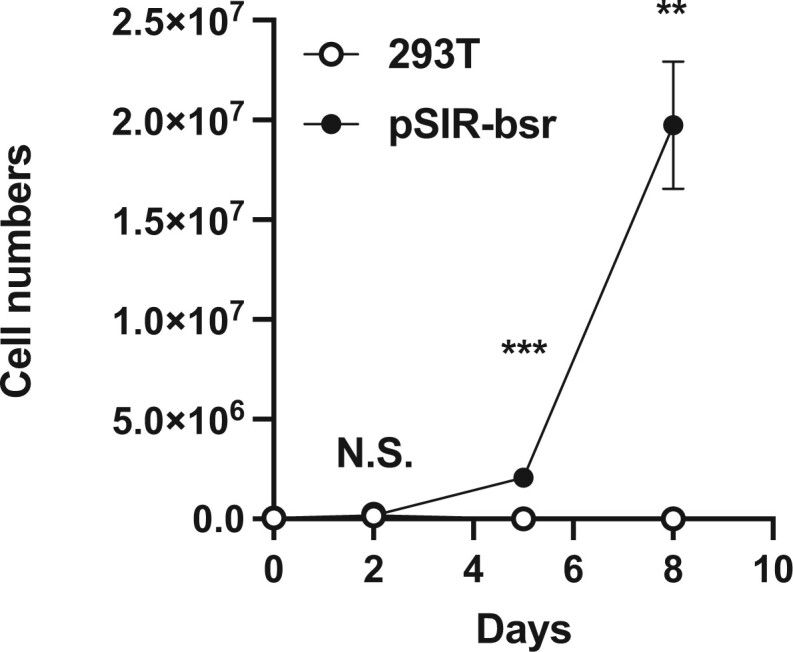
Proliferation of parental 293 T cells and 293 T cells transduced with pSIR-bsr in the presence of blasticidin S. Statistical significance was calculated using the *t*-test. The *P*-values for each time point were 0.9618 (Day 2), 0.0006 (Day 5) and 0.0035 (Day 8). This graph is a representative of the data of two independent experiments.

Next, proliferation of 293T and 293T cells transduced with pSIR-bsr was examined in the presence of 4.5 µg/ml blasticidin S. After plating in a six-well tissue culture dish (in triplicate), cell numbers were counted every 2 days, at which point 1 × 10^5^ cells were replated. As shown in Fig. 3, the parental 293T cells were completely killed by Day 5 after exposure to blasticidin S. In contrast, 293T cell transduced with pSIR-bsr continued to proliferate. These data show that integration of pSIR-bsr into the genome endowed the ability to proliferate in the presence of blasticidin S at a concentration sufficient to kill the parental cells.

It would be interesting to compare differences in the performances of pSIR (and its derivative pSIR-neo [6]) and pSIR-bsr. For example, there might be differences where these retrovirus vectors integrate into the genome, although at the present time there is no evidence to show that selection markers affect where retroviruses integrate into the genome. However, it is an interesting topic that will demand further investigation. In addition, pSIR- and pSIR-bsr-transduced cells might show differences in their rates of proliferation. Previously, I observed that when pSIR-neo was used, G418 selection was slower than blasticidin S selection, that is, parental cells survived longer in the presence of G418 than in the presence of blasticidin S. In this regard, since 293T cells acquire the neomycin-resistance gene during their establishment by transfection with SV40 T antigen, it is not possible to directly compare pSIR and pSIR-bsr using 293T cells. It seems unlikely that the proliferation profiles of cells transduced with pSIR and pSIR-bsr are different in the absence of selection drugs. Proliferation in the presence of drugs, however, may be different. However, this may depend on the concentrations of the selection drugs, and it is unlikely to depend on the integrated proviruses. Another possibility is that the expression of the neomycin-resistance gene or the *bsr* gene may impact cell proliferation by posing a burden on metabolic or other pathways. This possibility will be investigated in a future study.

Taken together, these results suggest that pSIR-bsr is useful for transducing cells with multiple expression vectors carrying different selection markers.

### Limitations

A limitation of the study is that I used only one cell line, 293T. However, since there are much data in the literature regarding the drug resistance of many different cell lines and primary cells, it would not be difficult to alter the experimental conditions (e.g. the transduction method and the concentration of blasticidin S used for selection) to enable the use of pSIR-bsr with other cell lines.

## Conclusions

I developed pSIR-bsr, a self-inactivating retrovirus vector retaining the *bsr* gene. To the best of our knowledge, Addgene contains no self-inactivating retrovirus vector expressing the *bsr* gene as a selection marker for use in mammalian cells. Therefore, this plasmid might give users greater flexibility when choosing the optimal drug for selecting transduced cells.
